# Changing pattern of suicide deaths in India

**DOI:** 10.1016/j.lansea.2023.100265

**Published:** 2023-08-17

**Authors:** Suryakant Yadav, Aathavan K K, Solveig Argeseanu Cunningham, Pravat Bhandari, Udaya Shankar Mishra, Aditi Aditi, Ravita Yadav

**Affiliations:** a Department of Bio-Statistics and Epidemiology, International Institute for Population Sciences (IIPS), Mumbai, India; b Hubert Department of Global Health, Rollins School of Public Health, Emory University, USA; c Department of Survey Research and Data Analytics, International Institute for Population Sciences (IIPS), Mumbai, India; d Department of Migration & Urban Studies, International Institute for Population Sciences (IIPS), Mumbai, India

Suicide is an untimely and avoidable death. It occurs within a societal, cultural, and familial context intertwined with human psychology. Given its proximate connection with mental health disorders, it is a public health concern. Suicide prevention is urgent owing to its repercussions for mortality and means of preventing self-harm. In 2015–2016, any psychological disorders were reported to affect 13.7% of population aged 18 years and older in India.^1^ Concomitantly, high suicide mortality among adults in India has become a public health concern.^1^^,^^2^ While India’s suicide rate of 14.04/lakh population in 2019 puts it at 49th rank globally, the grim reality of the highest numbers of suicides being reported annually from India cannot be overlooked.^3^

In the Lancet Public Health, Dandona et al.^4^ explored suicide deaths among Indian women by sociodemographic risk factors using National Crimes Record Bureau (NCRB)^5^ data. They found a trend of increasing suicide deaths among women with class 6 and higher education versus no education. The study reported a slight reduction in suicide deaths among married women but an uptick among never-married women. Housewives shared half of such eventualities that did not alter over time. Overall, a sociodemographic characteristic reading of suicide deaths among Indian women remained unchanged.

For comparison, we examined patterns of suicide death among Indian men and women. Analyses of unnatural deaths^6^ among adults (15–49 years) using National Family Health Survey data and suicide among 15 years and older based on the Million Death Study^2^ show that men more frequently die from suicide than women.

We retrieved data on suicide deaths from 2014 to 2021 from NCRB reports.^5^ While this is a crucial source of data, offering a characteristic description of suicide deaths by sociodemographic factors such as age, education, marital status, profession, economic status, means of suicide and reasons segregated by gender; however, underreporting of suicide deaths in NCRB compared to Global Burden of Disease has been highlighted.^7^ Suicide death rate (SDR)/lakh persons by age, education, marital status,^8^ and profession^9^ was calculated in 2014 and 2021 using NCRB data and NSS (2014, 2020–2021)^8^^,^^9^ and calibrated with projected population.

NCRB data for Indian men offers a change in the characteristic pattern of suicide deaths, unlike in the case of women. Findings indicate that SDRs in men compared to women were twice common in 2014, which increased to 2.5 times in 2021 (Table 1). The age group with the most suicide deaths for men were 18–29, 30–44, and 45–59 years, whereas for women were 18–29 years. Daily wage earners show a surge of 170.7% in suicide deaths among men between 2014 and 2021 accruing to SDR of 34.6 among men against 13.1 among women in 2021. Unemployed men and women had a very high SDR of 48.2 and 27.8, respectively. Women of all educational levels show a decline in suicide deaths, whereas men's suicide mortality increased at all educational levels. Most markedly, men who studied up to class 9–12 show a rise in SDR from 22.6 to 30.0 and an increase of 66.4% in suicide deaths between 2014 and 2021, approximately two-fold than their counterparts. The sex ratio of suicide deaths in the economic class of $1220–$6098 has risen from 2.3 to 3.7 times among men compared to women during 2014–2021. The suicide method frequently used was hanging, with an increase of 77.4% in men and 51.3% in women.

**Table 1:** Suicide death rate (SDR), ratio of male to female suicide deaths and the percentage change, India, 2014–2021.

Characteristics/Year	Suicide death rate (SDR) among men		Suicide death rate (SDR) among women		Ratio of male to female number of suicide deaths								Percent change in suicide deaths among men	Percent change in suicide deaths among women
	2014	2021	2014	2021	2014	2015	2016	2017	2018	2019	2020	2021	2014–2021	2014–2021
**Age group**														
10–18 years	5.1	4.7	5.8	6.0	1.0	0.9	0.9	0.9	0.9	0.9	0.9	0.9	−9.1	5.3
18–29 years	20.0	25.6	13.5	13.3	1.6	1.6	1.6	1.6	1.6	1.7	1.9	2.0	38.8	6.1
30–44 years	22.7	27.2	8.6	7.9	2.6	2.7	2.6	2.8	2.7	3.1	3.2	3.5	31.8	−0.8
45–59 years	19.9	23.7	5.9	5.6	3.5	3.6	3.5	3.9	3.7	4.2	3.9	4.4	33.2	6.8
Above 60 years	14.8	15.5	5.3	5.1	2.7	2.8	2.9	3.0	3.0	3.1	3.0	3.1	54.7	33.6
**Marital status**														
Never married	8.6	12.1	7.3	7.9	1.8	1.9	1.8	1.9	1.9	1.9	2.1	2.3	51.7	23.2
Currently married	20.0	24.3	8.7	8.4	2.2	2.3	2.3	2.4	2.3	2.6	2.6	2.8	35.7	6.0
Previously married	17.4	13.2	3.9	2.6	1.3	1.4	1.6	1.5	1.3	1.5	1.7	1.7	1.2	−23.8
Status not known/Others	–	–	–	–	2.5	2.7	2.3	1.8	2.1	2.5	2.6	3.0	−11.7	−25.1
**Education**														
Illiterate	12.8	13.5	4.1	3.5	1.7	1.8	1.6	1.9	1.9	1.9	2.0	2.1	4.0	−17.6
Class 1–5	11.0	12.2	6.4	5.4	1.9	2.1	2.1	2.2	2.1	2.4	2.4	2.4	11.1	−11.4
Class 6–8	18.1	22.5	12.0	10.6	2.0	2.2	2.2	2.3	2.4	2.5	2.5	2.7	28.4	−2.7
Class 9–12	22.6	29.9	13.7	14.2	2.4	2.4	2.3	2.3	2.2	2.5	2.6	2.9	66.4	37.7
Graduate & above	6.0	8.9	3.7	4.7	2.5	2.4	2.5	2.4	2.3	2.6	2.6	2.7	100.0	92.2
Status not known	–	–	–	–	2.2	2.3	2.0	1.9	2.1	1.9	2.0	2.6	−5.8	−20.1
**Profession**^b^														
House wife	–	–	–	7.0	–	–	–	–	–	–	–	–	–	15.0
Professionals/Salaried employee	–	18.0	–	9.0	5.4	7.1	7.5	6.6	7.5	7.5	6.7	8.1	70.7	13.9
Self-employed	–	19.0	–	10.6	7.8	10.9	11.0	10.7	12.1	8.6	12.6	14.0	26.4	−29.0
Student	–	3.3	–	3.4	1.1	1.1	1.0	1.1	1.1	1.2	1.3	1.3	73.6	49.5
Unemployed	–	48.2	–	27.8	5.9	6.1	4.8	5.2	4.8	4.8	4.7	5.9	38.2	38.7
Daily wage earner	–	34.6	–	13.1	7.8	6.0	6.7	7.0	7.5	8.4	7.4	8.9	170.7	137.1
Other	–	40.3	–	19.0	2.9	3.5	2.9	2.9	3.0	3.2	3.1	3.1	−42.2	−44.9
**Methods**														
Drowning	–	–	–	–	1.8	2.0	2.0	2.1	2.0	2.2	2.0	2.2	20.8	−1.9
Hanging	–	–	–	–	2.5	2.6	2.5	2.5	2.4	2.7	2.8	3.0	77.4	51.3
Insecticides consumption	–	–	–	–	2.2	2.4	2.3	2.3	2.3	2.4	2.2	2.5	94.9	66.7
Consumption of other poison	–	–	–	–	2.0	1.9	2.0	1.7	1.7	1.9	2.1	2.4	−22.7	−35.9
Jumping	–	–	–	–	4.2	4.5	4.7	4.8	4.0	5.0	4.4	5.3	24.2	−0.3
Other	–	–	–	–	2.2	2.3	2.0	2.4	2.2	2.2	2.0	2.0	−51.0	−46.6
Overdose of pills	–	–	–	–	1.6	1.6	1.8	2.1	1.7	1.8	1.6	1.9	9.3	−6.6
Self-immolation	–	–	–	–	0.6	0.6	0.7	0.5	0.6	0.6	0.7	0.7	−50.4	−56.3
**Reasons**														
Economic	–	–	–	–	8.0	7.1	6.6	10.4	9.2	9.4	10.0	9.9	93.7	57.7
Exam	–	–	–	–	1.3	1.3	1.2	1.3	1.2	1.4	1.2	1.5	−27.0	−34.7
Family problems	–	–	–	–	1.9	1.9	1.9	1.8	1.9	2.0	2.2	2.4	107.5	58.1
Health issues	–	–	–	–	2.5	2.5	2.7	2.9	3.1	3.4	3.1	3.2	59.4	25.1
Love affair	–	–	–	–	1.4	1.3	1.1	1.3	1.4	1.4	1.5	1.6	91.2	67.6
Marriage related problems	–	–	–	–	0.5	0.6	0.6	0.7	0.8	0.8	0.8	0.9	62.3	−7.8
Other	–	–	–	–	2.3	2.6	2.4	2.4	2.1	2.3	2.5	2.4	−56.8	−58.6
Personal/Social	–	–	–	–	2.5	2.6	2.7	2.6	2.7	3.0	3.1	3.0	107.9	74.6
Rape	–	–	–	–	–	–	–	–	–	–	–	–	a	−22.1
Unknown	–	–	–	–	2.3	2.2	2.4	2.3	2.1	2.5	2.4	2.8	3.7	−14.4
**Economic status (annual)**														
<$1220	–	–	–	–	2.0	2.0	1.9	2.0	2.0	2.1	2.1	2.3	19.0	5.8
≥$1220 & <$6098	–	–	–	–	2.3	2.6	2.7	2.5	2.6	3.0	3.3	3.7	64.7	3.4
≥$6098 & <$12,195	–	–	–	–	2.4	2.7	2.9	3.0	2.4	3.3	3.4	3.6	72.7	14.8
≥$12,195	–	–	–	–	2.7	2.2	9.7	2.0	2.9	2.8	2.6	2.1	51.9	98.1
**Total**	**17.0**	**20.6**	**8.5**	**8.1**	**2.1**	**2.2**	**2.1**	**2.2**	**2.2**	**2.4**	**2.4**	**2.6**	**33.5**	**5.9**

a Data on rape among men is small. The PPP is considered as $1 = 82 INR (as on July 2023). The re-categorisation of characteristics is the same as that of Dandona, George.^4^

b Data on population by profession is available in NSS (2020–2021)^9^ and so calculated for 2021 only.

Family problems and health issues remain the prominent reason behind suicides. Owing to these two reasons, the male-to-female ratio of suicides has increased from 1.9 and 2.5 to 2.4 and 3.2, respectively, during 2014–2021. There was a 107.5% increase in citing family problems as a reason among men during 2014–2021, approximately two-fold of that in women. The SDR among currently married men (24.3) was three times that of currently married women (8.4). Increases in suicide mortality were found in married and never married men, and the increase was remarkably higher than in women.

Rising suicides among married men and daily wage earners, having frequently cited reasons being family problems followed by health issues, are highly concerning. This increasing number of suicidal deaths needs further research to understand the nature of the stressors that trigger men to take the extreme step. Intervention strategies should focus on decriminalising and destigmatising suicide. Further, creating awareness of mental health issues in men would help arrest the increasing toll of suicides. Low suicide among women might indicate better coping mechanisms for dealing with stress,^10^ which could be an adaptable intervention strategy. A larger share of suicides in productive years of life calls for addressing livelihood insecurities.

## Contributors

SY conceptualised the study; AKK contributed to data extraction and curation; SY, AKK, and PB contributed to formal analysis; SY wrote the first draft of manuscript; SAC contributed to data interpretation and editing; USM, SY, and SAC revised the manuscript; AA, PB, and RY contributed in review and editing of the manuscript; All authors read and approved the manuscript.

## Declaration of interests

Suryakant Yadav was partially funded by COALESCE program supported by the Fogarty International Center of the National Institutes of Health (NIH) under Award Number D43 TW011404. We have not received any grant for this study. The authors declare no conflict of interest.
